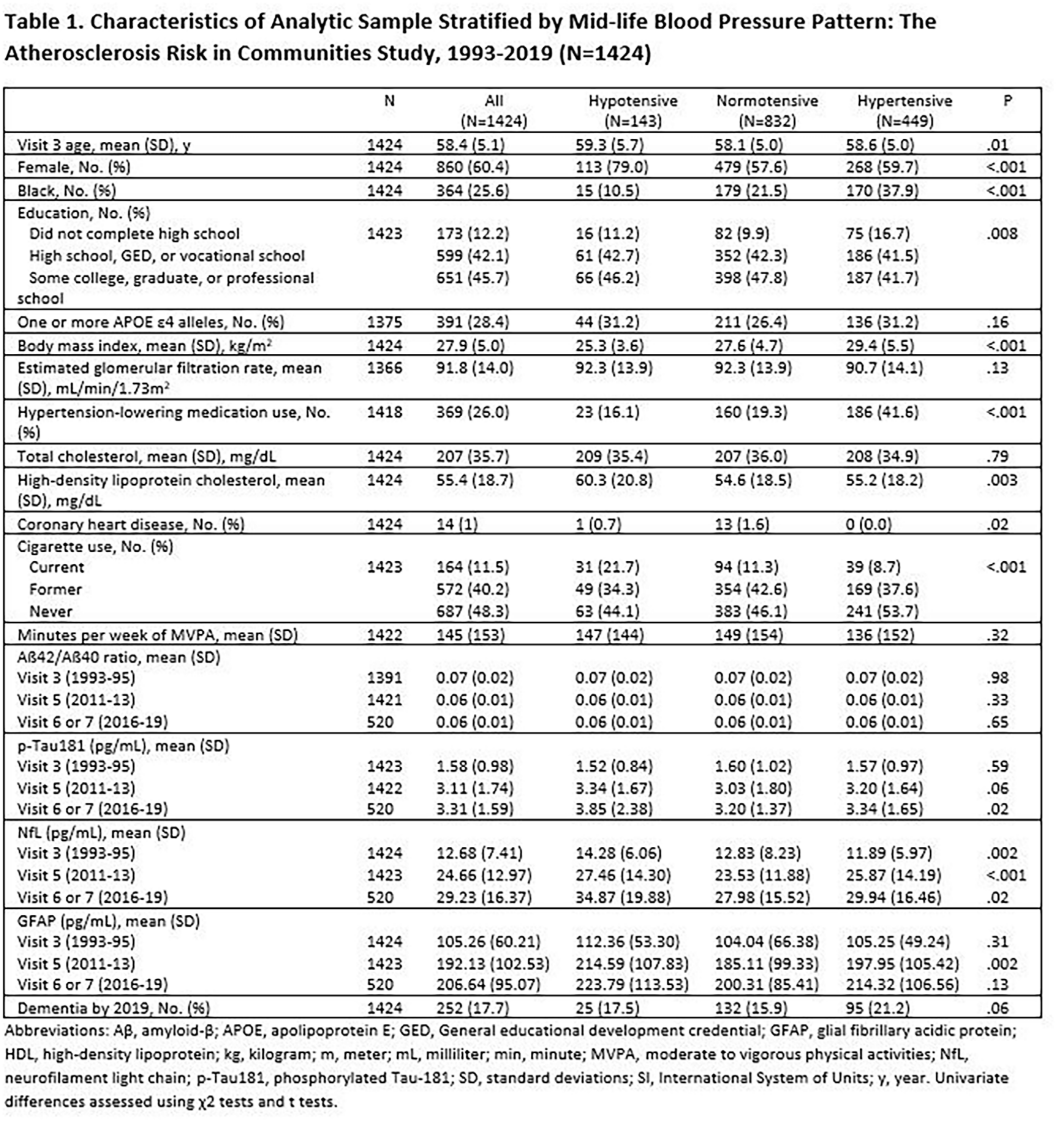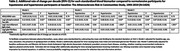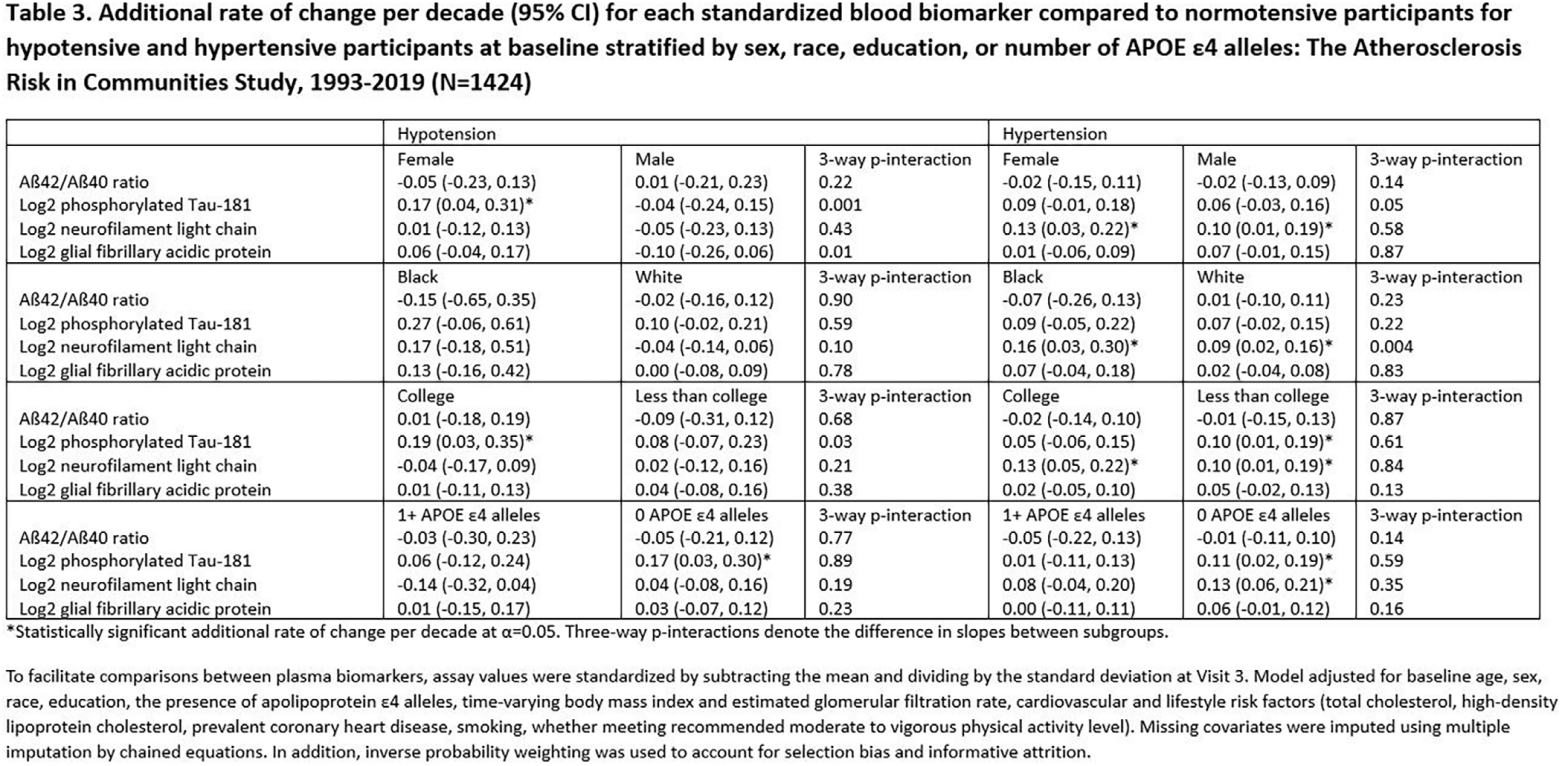# Association of mid‐life blood pressure status with longitudinal trajectories of blood‐based biomarkers of Alzheimer’s disease neuropathology and neurodegeneration in the Atherosclerosis Risk in Communities (ARIC) Study

**DOI:** 10.1002/alz.094902

**Published:** 2025-01-09

**Authors:** Jinyu Chen, James Russell Pike, Yifei Lu, Rebecca F. Gottesman, Keenan A. Walker, Zhengyi Gu, Michelle M. Mielke, Sanaz Sedaghat, Kevin J. Sullivan, Bharat Thyagarajan, Richey Sharrett, Thomas H. Mosley, Josef Coresh, Priya Palta

**Affiliations:** ^1^ University of North Carolina Chapel Hill, Chapel Hill, NC USA; ^2^ New York University, New York, NY USA; ^3^ National Institute of Neurological Disorders & Stroke Intramural Research Program, National Institute of Health, Bethesda, MD USA; ^4^ National Institute on Aging, National Institutes of Health, Baltimore, MD USA; ^5^ Wake Forest University School of Medicine, Winston‐Salem, NC USA; ^6^ University of Minnesota, Minneapolis, MN USA; ^7^ University of Mississippi Medical Center, The MIND Center, Jackson, MS USA; ^8^ Johns Hopkins University Bloomberg School of Public Health, Baltimore, MD USA; ^9^ University of Mississippi Medical Center, Jackson, MS USA

## Abstract

**Background:**

Current blood biomarkers of Alzheimer’s disease (AD) neuropathology and neurodegeneration include the ratio of amyloid‐β 42 to 40 (Aβ42/Aβ40), phosphorylated tau at threonine 181 (p‐Tau181), neurofilament light (NfL) and glial fibrillary acidic protein (GFAP). Prior studies have reported that hypertension is cross‐sectionally associated with lower levels of Aβ42/Aβ40 and longitudinally associated with faster accumulation of NfL. In this longitudinal investigation, we expanded on prior research by examining whether mid‐life blood pressure status was associated with change in AD biomarkers from mid‐ to late‐life.

**Method:**

In the Atherosclerosis in Communities Study (ARIC) cohort, 1424 participants had blood pressure measurements at Visit 3 (baseline, 1993‐95) and two or more measurements of AD blood biomarkers from Visit 3, Visit 5 (2011‐13), and Visit 6 or 7 (2016‐2019). Linear mixed effects models quantified the association of mid‐life blood pressure status (hypotension, SBP<90 or DBP<60; hypertension, SBP>130 or DBP>90; normotension) with the rate of change in each AD blood biomarker. Models were adjusted for age, sex, race, education, the presence of apolipoprotein ε4 (APOE ε4) alleles, time‐varying body mass index and estimated glomerular filtration rate, and baseline cardiovascular and lifestyle risk factors.

**Result:**

The sample included 860 women (60.4%) and 364 Black participants (25.6%). At baseline, 143 participants (10.0%) had hypotension and 449 (31.5%) had hypertension. Compared to participants with normotension, mid‐life hypertension was associated with accelerated accumulation of NfL from mid‐ to late‐life (Table 2). Both mid‐life hypotension and hypertension were associated with accelerated accumulation of pTau‐181 from mid‐ to late‐life. In subgroup analyses, these associations were greater among women, Black participants, and individuals without APOE ε4 alleles.

**Conclusion:**

Mid‐life hypotension and hypertension are associated with faster changes in both AD‐specific (pTau‐181) and neurodegenerative (NfL) biomarkers from midlife to late‐life. Future investigation is needed to determine whether hypotension and hypertension are on the causal pathway relating AD neuropathology to dementia or if abnormal blood pressure and faster accumulation of pTau‐181 are shared characteristics of other comorbidities.